# The Utility of a Three-gene Host Response to Discriminate Tuberculous Meningitis From Other Infections in Children

**DOI:** 10.1097/INF.0000000000005105

**Published:** 2025-12-23

**Authors:** Julie Huynh, Nhat Hoang Thanh Le, Bao Hoai Le Nguyen, Hai Thanh Hoang, Van La Ngoc, Samuel Ensor, Khanh Quoc Nguyen Phan, Ny Hong Thi Tran, Tram Ngoc Pham, Thu Anh Dang Do, Trinh Thi Bich Tram, Dung Thi Mong Vu, Vinh Dinh Do, Anna Griffiths, Suzanne Anderson, Diana Gibb, Dang Minh Thi Ha, Trinh Huu Tung, Nguyen Dinh Qui, Nguyen Hong Thi Nhung, Guy E. Thwaites, Nguyen Thuy Thuong Thuong, on behalf of the SURE trial team

**Affiliations:** From the * Oxford University Clinical Research Unit, Ho Chi Minh City, Vietnam; † Nuffield Department of Medicine, Centre for Tropical Medicine and Global Health, Oxford University; ‡ NOTSSCaN Division, Oxford University Hospital NHS Trust, Oxford; § Institute of Clinical Trials and Methodology, Medical Research Council Clinical Trials Unit at University College of London, High Holborn, United Kingdom; ¶ Department of Paediatrics, Pham Ngoc Thach Hospital for Tuberculosis and Lung Disease; ‖ Department of Infectious Diseases, Children’s Hospital 2, Ho Chi Minh City, Vietnam.

**Keywords:** child, tuberculous meningitis, diagnostic, transcriptomic

## Abstract

**Background:**

Early diagnosis of tuberculous meningitis (TBM) is critical to favorable outcomes. We investigated whether a 3-gene host response signature in whole blood can distinguish TBM from symptomatic controls in children.

**Methods:**

Whole-blood RNA sequencing was performed in children with TBM and controls. Expression of the 3-gene signature, [guanylate-binding protein (GBP5), dual specificity phosphatase 3 (DUSP3) and Krupple-like factor 2 (KLF2)] was quantified and a tuberculosis (TB) score was calculated using (GBP5+DUSP3)/2-KLF2. Discriminatory performance was obtained using receiver-operator characteristic curve analysis against microbiologic and composite reference standards. TB score and 3-gene expression in children were compared against adults with TBM. In parallel, an exploratory transcriptome-wide analysis was performed, applying bootstrapped least absolute shrinkage and selection operator regression to identify additional genes associated with TBM.

**Results:**

Forty-two children had TBM and 41 were controls. KLF2 was upregulated in TBM compared to controls (*P* = 0.043); while GBP5, DUSP3 and TB score showed no difference. The diagnostic performance of GBP5 alone (area under the curves: 0.64; 95% confidence interval: 0.46–0.83) and TB score (area under the curves: 0.59; 95% confidence interval: 0.41–0.77) was poor against the reference standard of definite TBM. GBP5 in children with TBM was lower than in adults without HIV (median 13.04; interquartile ranges: 11.91–14.29 vs. median 13.72; interquartile ranges: 12.58–14.53, *P* = 0.036), and expression was nonlinear across the age spectrum; lowest in young children. Exploratory transcriptomic analysis suggests that novel genes may contribute a discriminatory signal.

**Conclusion:**

The 3-gene host response signature does not discriminate TBM from controls in children and was much less discriminative compared to adults. An alternative set of pediatric-specific signatures may exist, but further discovery and validation are required.

Tuberculous meningitis (TBM), the most devastating form of tuberculosis (TB), disproportionately affects young children.^1^ Mortality from TBM remains high (20%–50%)^2,3^ and over half of childhood survivors have long-term disability.^1,3^ Poor outcomes in children are strongly associated with delayed diagnosis, due to presentation with nonspecific symptoms, and the low sensitivity of diagnostic tests such as Xpert MTB/RIF assay (Xpert; Cepheid, Sunnyvale, CA) pooled sensitivity 0.42 [95% confidence interval (CI): 0.22–0.63]; pooled specificity 0.99 (95% CI: 0.95–1.00).^4,5^ Xpert/Ultra, the World Health Organization (WHO) recommended initial diagnostic test for all forms of TB, is not widely available in local health care settings where children make their first health encounter.^6,7^ On average children have 4 healthcare visits before a diagnosis of TBM is made,^8^ further complicated by complex referral pathways.^9,10^

The WHO promotes the development of child-friendly, and nonsputum point-of-care tests for pulmonary TB (PTB) and extrapulmonary TB (EPTB).^11^ The 3-gene Xpert-MTB-Host response (MTB-HR) prototype (Cepheid, Sunnyvale, CA) is a point-of-care test that uses only 100 μL of blood, offering practical utility in children. In a large multicenter study of presumptive pediatric TB (mostly PTB) Xpert-MTB-HR differentiated culture confirmed TB from unlikely TB with 60% sensitivity (95% CI: 50.8–68.4) and 42% sensitivity (95% CI: 34.7–48.7) against any microbiological reference standard, with 90% specificity.^12^ Combined Xpert-MTB-HR with one Xpert MTB/Rif Ultra identified 71% of microbiologically confirmed cases, suggesting potential value for inclusion in the current diagnostic armamentarium.^12^ In adults, Xpert-MTB-HR shows higher accuracy 80% sensitivity (95% CI: 76%–85%), 94% specificity (95% CI: 91%–96%) than in children.^13^ However, data from children with EPTB remains very limited, suggesting diagnostic accuracies below WHO’s 2024 target product profile for pediatric EPTB.^14^

Whole blood analysis offers advantages over cerebrospinal fluid (CSF) collection, particularly in young children, where recommended minimum CSF volumes are difficult to obtain.^15^ A simple fingerstick blood test, applicable to TBM, has potential as a stand-alone or ancillary diagnostic. We previously demonstrated, as proof of concept, that a 3-gene host response signature [guanylate binding protein (GBP5), dual specificity phosphatase 3 (DUSP3) and Krupple-like factor 2 (KLF2)] discriminated TBM from other brain infections in adults.^16^ Expression of these genes combined, forms a minimal signature; reflecting the balance between interferon-driven immune activation (GBP5 upregulation) and immune dysregulation (DUSP3 upregulation and KLF2 downregulation) in active TB.^17^ Here, we assessed its diagnostic performance in children with suspected TBM and conducted an exploratory transcriptomic analysis to identify differentially expressed genes in those with and without TBM.

## METHODS

### Participant Cohorts

The first cohort consisted of children and adolescents (29 days to <18 years old) with signs and symptoms of a brain infection who were screened for enrollment in a randomized controlled trial of antituberculosis and anti-inflammatory therapy [short intensified treatment for children with TBM (SURE trial); ISRCTN40829906] at Pham Ngoc Thach Hospital and Children’s Hospital 2, Ho Chi Minh City, Vietnam^18^ from March 2021 to September 2022. Those without TBM were controls and had separate consent taken for the acquisition of samples and clinical information.

Children with TBM, defined by clinical features and compatible CSF with or without microbiological confirmation, were retrospectively classified into definite, probable and possible TBM using published criteria.^19^ Definite TBM required at least 1 positive CSF test, Ziehl Neelsen stain, Xpert Ultra or mycobacterial culture (mycobacterial growth incubator tube). Probable and possible TBMs were unconfirmed and classified by a point scoring system using the uniform case definition for research^19^ (Table, Supplemental Digital Content 1, https://links.lww.com/INF/G464). TBM disease severity was graded using British Medical Research Council definitions (Table, Supplemental Digital Content 2, https://links.lww.com/INF/G464). The group TBM refers to all cases (definite/probable/possible) unless stated otherwise. All participants with TBM were tested for HIV.

Children with suspected non-TBM infection were enrolled as controls if they had a confirmed alternative diagnosis or presumptive brain infection with clinical improvement without antituberculosis therapy (ATT). The “recovery without ATT,” was a pragmatic approach to defining non-TBM controls, as TBM is almost always fatal without ATT. Controls could be reclassified as TBM if later diagnosed and initiated on ATT. Standard-of-care diagnostics followed local hospital guidelines, including blood cultures, CSF gram stain and bacterial culture. Parasitic serology and polymerase chain reaction, fungal culture, limited viral polymerase chain reaction testing and Japanese encephalitis IgM were performed when clinically indicated. HIV testing was not mandatory in controls and performed only when clinically indicated. Controls were later subclassified into non-CNS infections (normal CSF) and meningoencephalitis.

The second cohort included adults with TBM, from 2 randomized controlled trials (ACT and LAST ACT),^20,21^ and adults with other brain infections from an observational study.^16^

### Blood Sample Collection and RNA-sequence Preprocessing

Venous blood was collected from all participants at enrollment, at the same timepoint as CSF samples for Ziehl Neelsen, Xpert MTB ultra and mycobacterial growth incubator tube. Samples were collected in PAXgene Blood RNA tubes (PreAnalytiX, Hombrechtikon, Switzerland), stored at −80 °C with RNA extracted as previously described.^16^ Libraries were generated on the Sciclone G3 NGS (Perkin Elmer, UT), pooled and sequenced using NovaSeq 6000 S4 reagents at 2 × 100 bp to generate ~30 million reads per sample. RNA sequencing (RNA-seq) on pediatric samples was performed in 1 batch.

Quality control of RNA-seq data was performed using an in-house pipeline derived from published analysis.^22,23^ Gene counts were generated from sequencing fastq files. Log2 scale transformations were performed and normalized for library size using DESeq2 functions (DESeq2 package).^24^ Data on GBP5, DUSP3 and KLF2 were extracted for the main analysis and expressed as normalized gene expression. TB score (combined up and downregulated gene expression) was calculated as (GBP5 + DUSP3)/ 2 – KLF2.^17^ Post hoc analysis replacing KLF2 with tuberculin-binding-protein (TBP) was done to align with manufacturer instructions for the updated late-prototype of Xpert-MTB-HR.^25^ RNA-seq analysis was performed by statisticians and bioinformaticians blinded to clinical information and reference standard results. Clinical and index test data were not available to laboratory technicians performing TB diagnostics.

### Sample Size

Sample size calculation used technical and biological variability as previously described.^26^ We used a technical variation of (1/μ = 1/16) where μ is sequencing depth and a biological variation (cv^2^ = 0.4^2^) where cv is the coefficient of variation. To achieve 80% power with a 2-sided 5% significance level to detect 1.3-fold difference between 2 groups, a total of 50 participants per group were required to test for biomarker discovery.

### Statistical Analysis

The adult cohort has been previously described.^16^ The reported analyses relate to children only unless otherwise stated.

Patient characteristics were compared between TBM and symptomatic controls (non-TBM). Continuous variables are reported with medians, interquartile ranges (IQR, Q1–Q3). and categorical variables are presented as the number of cases and percentages.

Gene expression was compared using Mann–Whitney *U* test in pairwise analyses: TBM versus symptomatic controls, TBM only versus TBM with PTB subgroups and children versus adults with TBM. The diagnostic performance of GBP5 and TB score for distinguishing definite, definite/probable, and all TBM from controls was assessed using the area under the receiver operating characteristic curves (AUC). AUC for GBP5 was also stratified by age groups.

 The impact of PTB status on GBP5 expression and TB score was assessed with Mann–Whitney *U*. To assess the impact of age, generalized additive models assessed nonlinear association on combined pediatric and adult cohorts. We conducted an exploratory transcriptome-wide analysis to identify pediatric-specific genes that distinguished TBM from non-TBM. Given under-recruitment in both study arms, the prespecified fold-change threshold for differential expression was increased to a more conservative 1.5. Genes with notable differential expression between TBM and non-TBM were entered into a least absolute shrinkage and selection operator (LASSO) model^27^ bootstrapped 10,000 times to identify predictors of TBM status. Selection frequency was tallied across bootstraps, and AUCs were calculated for the most frequently selected gene.

All analyses were conducted using R version 4.2.2. False discovery rate was minimized by correcting for multiple testing using the Benjamini-Hochberg method.

## RESULTS

### Cohort Characteristics

A total of 83 children participated in this study (42 with TBM and 41 non-TBM controls). There were 27 (64%) and 27 (66%) males in the TBM and control groups, respectively. The median age was 2.4 years (IQR: 0.6–9.2) in children with TBM, and 3.0 years (IQR: 0.6–8.8) in controls (Table 1). No children in the TBM group were HIV positive. Four controls were tested for HIV, one of whom was positive. Median fever duration at presentation was significantly longer in children with TBM (13 days; IQR: 6–21) compared to controls (4 days; IQR: 3–12) (*P* = 0.003).

**TABLE 1.** Baseline Characteristics in Children With Tuberculous Meningitis and in Controls

Participant Characteristics	TBM (N = 42)	Controls (N = 41)	*P*-value
Age (median, Q1, Q3)	2.4 (0.6, 9.2)	3.0 (0.6, 8.8)	>0.99
Weight for age z-score (median, Q1, Q3)*	−0.76 (−1.74, 0.32)	−0.21 (−0.93, 0.37)	0.30
Female	15 (36%)	14 (34%)	0.88
BCG vaccinated	34 (81%)	37 (90%)	0.23
HIV status			0.09
Negative	41 (100%)	3 (75%)	
Positive	0 (0%)	1 (25%)	
Unknown†	1	37	
Fever	40 (95%)	39 (95%)	>0.99
Fever duration (days)	13 (6, 21)	4 (3, 12)	**0.003**
Glasgow coma score	15.00 (15.00, 15.00)	15.00 (15.00, 15.00)	0.49
Cranial nerve palsy	6 (14%)	1 (2.4%)	0.11
Hemi/para/tetraplegia	9 (21%)	4 (9.8%)	0.14
British MRC grade			
GRADE I	26 (62%)	–	
GRADE II	12 (29%)	–	
GRADE III	4 (9.5%)	–	
TBM classification			
Definite	19 (45%)	–	
Probable	10 (24%)	–	
Possible	13 (31%)	–	
Microbiologically confirmed pulmonary TB			
Negative	32 (78%)	41 (100%)	
Positive	9 (22%)	0 (0%)	
Unknown†	1	0 (0%)	

CSF Characteristics	TBM (N = 42)	Controls (N = 41)	*P*-value
Leukocyte count (cells/μL)	145 (52, 259)	44 (8, 148)	**0.002**
Neutrophils (%)	1 (0, 16)	2 (0, 10)	0.84
Unknown†	4	0	
Lymphocytes (%)	87 (70, 95)	70 (0, 96)	**0.024**
Eosinophils (%)	0.00(0.00, 0.00)	0.00 (0.00, 0.00)	0.60
Protein level (g/L)	1.40 (0.67, 1.87)	0.48 (0.25, 0.92)	**<0.001**
Glucose level (mmol/L)	1.85 (1.52, 2.66)	3.00 (2.70, 3.70)	**<0.001**
CSF/Blood glucose ratio	0.36 (0.25, 0.43)	0.71 (0.59, 0.80)	**<0.001**
Unknown†	2	3	

Blood Parameters	TBM (N = 42)	Controls (N = 41)	*P*-value
Hemoglobin (g/dL)	10.90 (9.30, 11.40)	10.80 (9.80, 12.60)	0.45
Leukocyte count (×10^3^ cells/μL)	12 (10, 16)	12 (10, 17)	0.96
Neutrophils (%)	51 (35, 66)	52 (30, 73)	0.89
Lymphocytes (%)	30 (20, 43)	33 (17, 56)	0.90
Platelet (×10^3^/μL)	404 (352, 531)	391 (291, 463)	0.11
Sodium (mmol/L)	134.0 (131.0, 137.0)	135.0 (134.0, 139.0)	0.10
Unknown†	1	5	

Symptomatic controls consisted of 28 non-TBM meningoencephalitis, 13 non-CNS infection. Non-TBM meningoencephalitis consisted of 5 microbiologically confirmed, 23 presumed. Non-CNS infection consisted of febrile seizure, headache of unknown cause, sepsis, macrophage activation syndrome, subdural hematoma, inborn error of metabolism, cerebral palsy and bacteremia, HIV, congenital hydrocephalus with intercurrent infection, lumbar spinal abscess, viral illness and urinary tract infection. *P* < 0.05 is considered statistically significant and is represented by values in bold.

* z-score = WHO standardized.

† Unknown due to missed sample collection by error, death or laboratory error.

BCG indicates Bacille Calmette-Guerin; CSF, cerebrospinal fluid; MRC, Medical Research Council; TBM, tuberculous meningitis.

Most children with TBM had Medical Research Council Grade 1 disease (62%). Nineteen (45%) had definite TBM, 10 (24%) had probable TBM and 13 (31%) had possible TBM. CSF pleocytosis was more prominent in children with TBM than controls (TBM: 145 cells/μL; IQR: 52–259, vs. controls: 44 cells/μL; IQR: 8–148; *P* = 0.002). The proportion of CSF lymphocytes was higher (87%; IQR: 70–95 vs. 70%; IQR: 0–96; *P* = 0.024) and CSF glucose and CSF/blood glucose ratio were both significantly lower compared to controls (*P* < 0.001 for both). CSF protein was significantly higher in children with TBM (1.40 g/L; IQR: 0.67–1.87 vs. 0.48 g/L; IQR: 0.25–0.92; *P* < 0.001) (Table 1).

The 41 controls consisted of 28 with meningitis or encephalitis other than TBM (5 with a confirmed pathogen, and 23 with presumptive bacterial meningitis/viral meningitis, encephalitis of unclear cause, eosinophilic meningitis or cerebral abscess) and 13 without CNS infection (Table, Supplemental Digital Content 3, https://links.lww.com/INF/G464**).** Microbiologically confirmed CNS infections were due to *Streptococcus pneumoniae* (n = 2), *Escherichia coli* (n = 1), Herpes Simplex Virus (n = 1) and Japanese encephalitis (n = 1).

Children with TBM received a median of 3 days (IQR: 1.0–4.8) ATT before enrollment, whilst controls received 3 days (IQR: 2.0–5.0) of other antibacterial drugs. Most children with TBM [29 (69%)] were enrolled before commencing corticosteroid therapy. Those who received corticosteroids had on average 2.8 days before enrollment. No child in the control group received corticosteroids. Median hospital stay was 14 days (IQR: 13–15) in children with TBM and 11 days (IQR: 6–16) in controls.

### 3-Gene Host Response Signature: TBM Versus Controls

**FIGURE 1.**
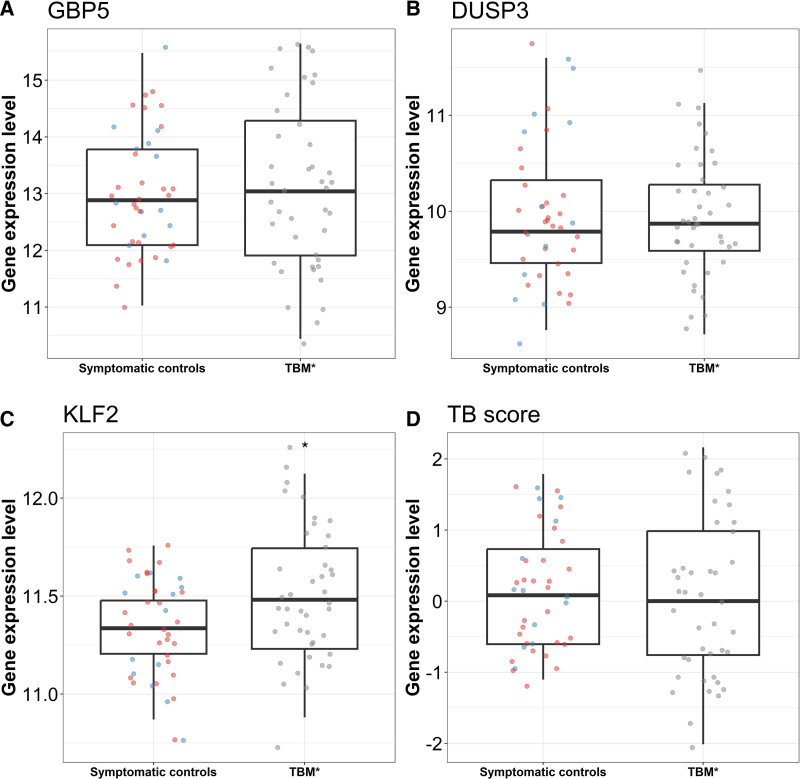
Three-gene expression and TB score in TBM compared to controls. A: GBP5, (B) DUSP3, (C) KLF2 and (D) TB score. Comparisons of gene expression in TBM (n = 42) and symptomatic controls (n = 41). TBM* = all TBM. Controls were classified into other meningitis/encephalitis (n = 28, red dots) or noninfectious (n = 13, blue dots). Boxes indicate interquantile range, the horizontal line indicates the median and dots indicate individual participant data. Comparisons were performed using Mann–Whitney *U* test. *P < 0.05.

There was no significant difference in GBP5 (*P* = 0.89) or DUSP3 (*P* = 0.85) gene expression between TBM and controls (Fig. 1). KLF2 expression was higher in TBM than controls (11.48; IQR: 11.23–11.74 vs. 11.34; 11.21–11.48; *P* = 0.043). However, this did not translate to a difference in TB score (*P* = 0.63). TBP expression did not differ between TBM and controls (*P* = 0.51) and nor did TB score when TBP replaced KLF2 (*P* = 0.75) (Figure, Supplemental Digital Content 4, https://links.lww.com/INF/G464). There was no significant difference in 3-gene host expression and TB score between other meningitis/encephalitis and non-CNS infection controls (Figure, Supplemental Digital Content 5, https://links.lww.com/INF/G464). Corticosteroid therapy did not influence comparisons of 3-gene host expression or TB score between TBM and controls (Figure, Supplemental Digital Content 6, https://links.lww.com/INF/G464).

### 3-Gene Host Response Signature in Children Compared to Adults

**FIGURE 2.**
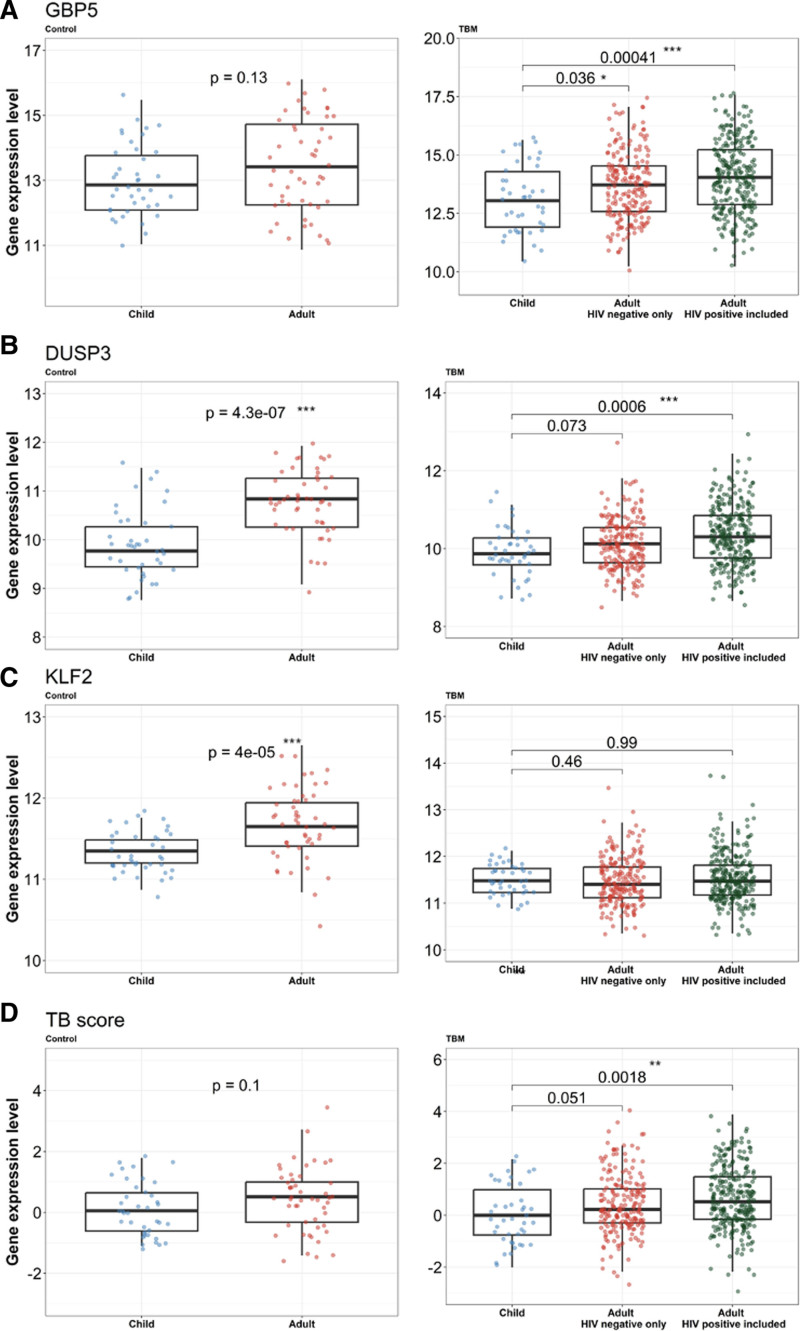
Three-gene expression and TB score: childhood TBM and controls compared to adults. A: GBP5, (B) DUSP3, (C) KLF2 and (D) TB score. Each panel illustrates graphs comparing child and adult control (left) and child and adult TBM (right). Comparisons of gene expression in childhood TBM (n = 42) versus adult TBM including HIV (n = 281) versus adult TBM without HIV (n = 207) and child (n = 40) versus adult controls (n = 50). Boxes indicate interquantile range, the horizontal line indicates the median and dots indicate individual participant data. Comparisons were performed using Mann–Whitney *U* test. *P < 0.05, **P < 0.01 and ***P < 0.001.

The adult cohort (n = 281) consisted of 207 with and 74 without HIV infection. As previously reported, 134 (48%) had definite, 98 (35%) had probable and 49 (17%) had possible TBM (Table, Supplemental Digital Content 7, https://links.lww.com/INF/G464).^16^ The 3-gene host expression was significantly reduced in children compared to adults (Fig. 2), with lower GBP5 (13.04; IQR: 11.91–14.29 vs. 14.04; IQR: 12.88–15.23; *P* < 0.001) and lower DUSP3 expression (9.87; IQR: 9.59–10.28 vs. 10.31; IQR: 9.76–10.85; *P* < 0.001). KLF2 expression was similar across groups (*P* = 0.99). TB score in adults was significantly higher than in children with TBM (0.52; IQR: 0.15–1.49 vs. 0.00; IQR: 0.76–0.99; *P* = 0.002).

As HIV coinfection was a confounder in our previous adult study,^16^ we compared 3-gene host expression in HIV-negative children and adults with TBM. GBP5 expression remained significantly lower in children (Fig. 2).

### Diagnostic Performance of 3-Gene Host Response Signature in Children

**FIGURE 3.**
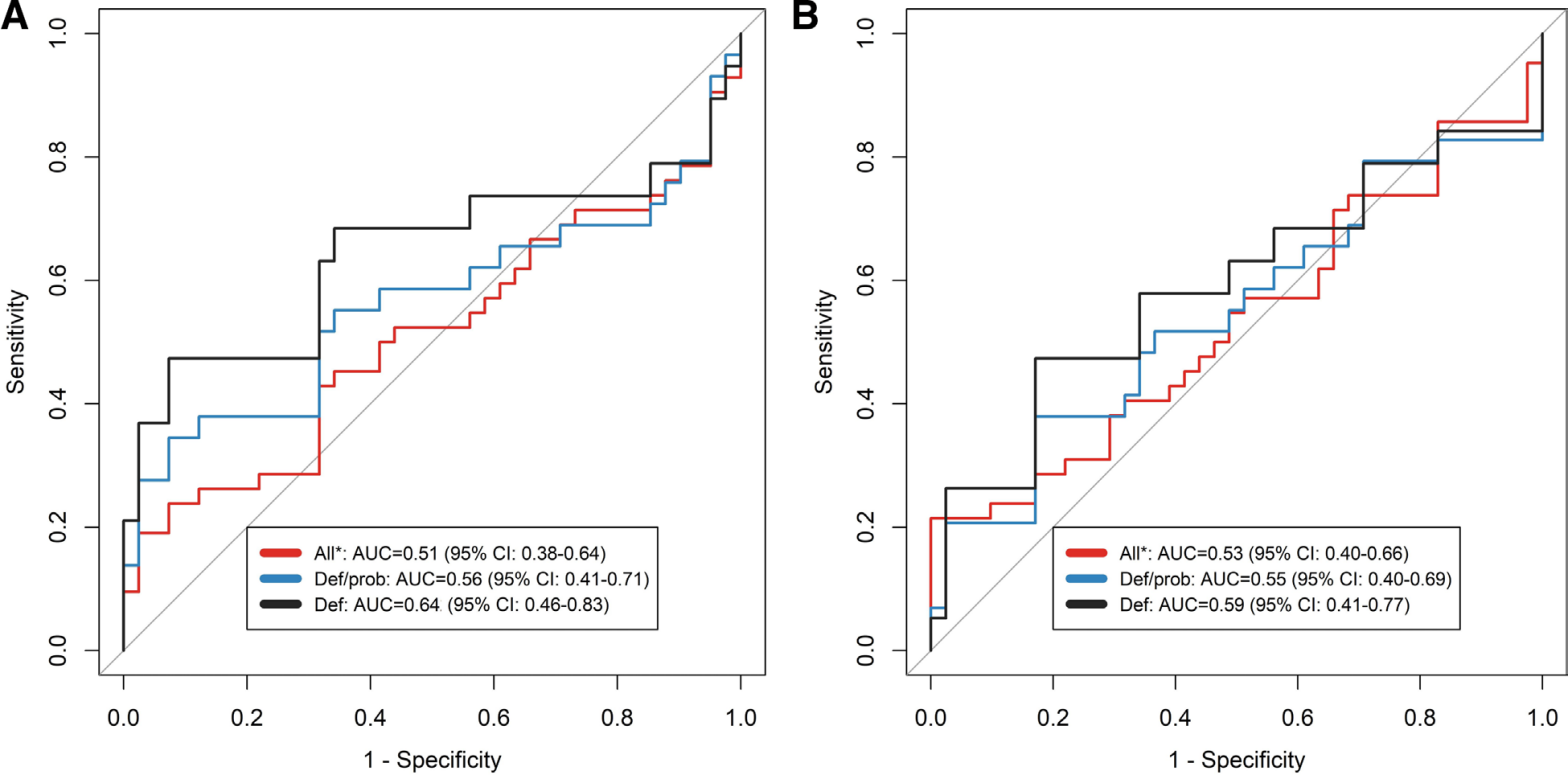
Diagnostic performance of GBP5 and TB score in TBM. Receiver operating characteristics (ROC) curves for distinguishing all TBM (n = 42), definite + probable TBM (n = 29) and definite TBM (n = 19) against symptomatic controls (n = 41) using GBP5 alone (A) or TB score (B).

The ability for GBP5, the most prominently expressed gene of the 3-gene host response signature, to discriminate between TBM and controls was poor. The AUC was 0.51 (95% CI: 0.38–0.64) in all TBM and 0.64 (95% CI: 0.46–0.83) in definite TBM (Fig. 3A). The TB score was a similarly poor diagnostic discriminator with an AUC of 0.53 (95% CI: 0.41–0.66) (Fig. 3B). Replacing KLF2 with TBP, did not improve TB score performance (Figure, Supplemental Digital Content 8, https://links.lww.com/INF/G464).

### Influence of Age on GBP5 Expression

**FIGURE 4.**
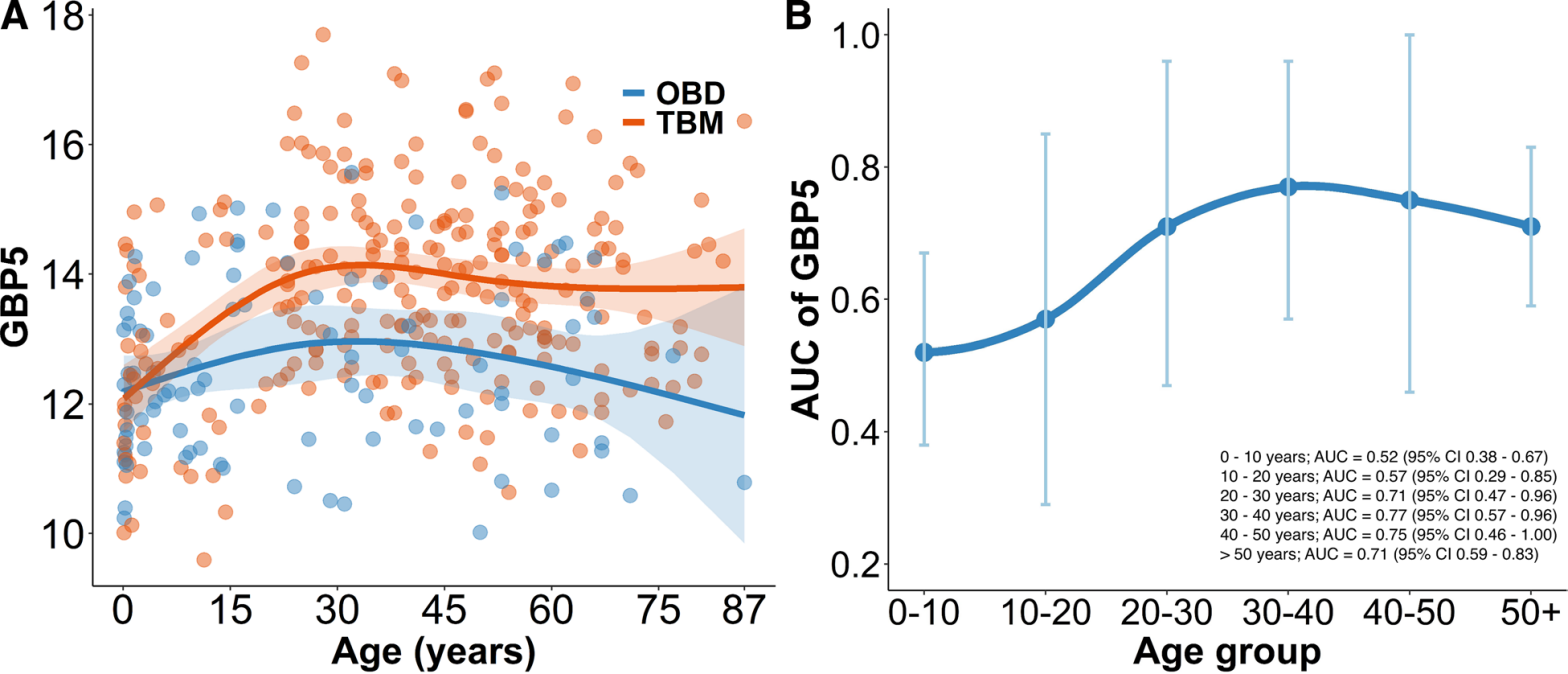
Combined age analysis of GBP5 expression. Each dot represents individual participant data from adults and children. Solid blue line represents line of best fit for other brain infections (OBD), which were adult (50) and child (41) controls. Solid orange line represents line of best fit for TBM all TBM without HIV infection (42 child, 207 adult) after generalized additive modeling. (A) GBP5 expression across the age spectrum (B) AUC of GBP5 according to age.

Combined analysis of children with adults demonstrated an age-related trend for GBP5 expression between TBM and controls. In TBM, GBP5 expression increased more steeply in early life compared to controls. This divergence became more pronounced with increasing age. After 30 years of life, GBP5 expression reached a plateau in both TBM and controls (Fig. 4A). The AUC for GBP5 in children (0–10 years) was 0.52 (95% CI: 0.38–0.67), lower than in adults 30–40 years of age (0.77; 95% CI: 0.57–0.96) (Fig. 4B).

### 3-Gene Host Response Signature: TBM Versus TBM With Lung TB

We compared the host response signature in children with TBM and lung TB (n = 25) to TBM only (n = 16). Lung TB was defined as the composite reference standard (microbiologically confirmed and presumptive PTB based on clinical features and chest radiograph, including miliary TB). DUSP3 was significantly higher in TBM with lung TB compared to TBM only (10.18; IQR: 9.85–10.50 vs. 9.77; IQR: 9.53–10.09, *P* = 0.006) (Figure, Supplemental Digital Content 9, https://links.lww.com/INF/G464). Conversely, KLF2 expression was significantly lower in TBM with lung TB (11.33; IQR: 11.21–11.3 vs. 11.57; IQR: 11.25–11.79, *P* = 0.023). This resulted in a TB score being higher in children with TBM and lung TB compared to TBM only (1.18; IQR: 0.59–1.01 vs. −0.09; 0.80–0.49, *P* = 0.028).

### Exploring Novel Gene Host Response Signatures for Childhood TBM

**FIGURE 5.**
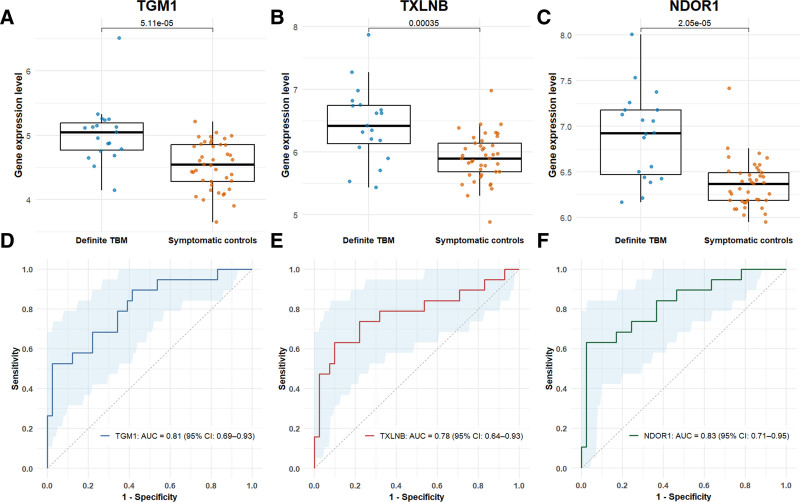
Performance of the top 3 differentially expressed genes in children with definite TBM compared with other infections. A–C: Gene expression level of the top 3 LASSO-selected genes (TGM1, TXLNB and NDOR1) in children with definite TBM (n = 19) compared with other infections (n = 41). Boxes show the interquartile range (IQR), horizontal lines show median and whiskers extend to 1.5 × IQR. Group comparison was performed using the Mann–Whitney *U* test (significance, *P* < 0.05). D–F: Area under the curve (AUC) for each of the top 3 LASSO-selected genes illustrating the ability to discriminate TBM from other infections.

Of the 13,355 protein-coding genes that showed stable expression across samples, a total of 959 genes met thresholds for differential expression (787 upregulated, 172 downregulated) (Figure, Supplemental Digital Content 10, https://links.lww.com/INF/G464). The top genes selected by the exploratory LASSO model were TGM1, TXNLB and NDOR1; an extended list of frequently selected genes is available in Table, Supplemental Digital Content 11, https://links.lww.com/INF/G464). Across these top genes, expression differed significantly between definite TBM and controls (*P* < 0.001, Fig. 5A–C), with individual AUC ranging between 0.78 and 0.83 (Fig. 5D–F).

## DISCUSSION

Early and accurate TBM diagnosis remains a major obstacle to improving outcomes. This is the first study to investigate whether a 3-gene host response signature in whole blood can discriminate TBM from other infections in children and to compare against performance in adults. We found that the 3-gene host response signature performed poorly in children. Gene expression was influenced by age and the presence of lung TB. Our findings suggest there are different host immune responses to *M. tuberculosis* in children and adults with TBM and that transcriptomic analysis in blood can be used to discover pediatric-specific signatures with improved diagnostic performance.

We demonstrated poor diagnostic performance of TB score and GBP5 across all TBM diagnostic reference standards. Substituting KLF2 for the more stable TBP, consistent with the latest Xpert-MTB-HR prototype, did not improve TB score. In contrast, our previous proof-of-concept study in adults showed that a 3-gene host response signature could discriminate TBM from other brain infections and that GBP5 alone was more discriminatory than the combinatory TB score.^16^ This discordance between populations is not unexpected, as the 3-gene host response signature was originally derived from a multicohort analyses of predominantly adult data.^17^ Subsequent studies evaluating the diagnostic performance of the same 3-gene host response signature using Xpert-MTB-HR also showed better performance in adults than children^12,13^; highlighting that performance in adults cannot be presumed to translate to children.

A large meta-analysis including whole blood transcriptomic profiles from African, European and South American adults and children with acute and latent TB, demonstrated child-specific upregulated genes.^28^ Gene expression profiles among Indian children with confirmed TB were also distinct from adult-derived gene lists.^29^ We demonstrated, using combined pediatric and adult data, that the difference in adult and child 3-gene host response signature could in part be explained by age-dependent GBP5 expression. Young children had lower GBP5 expression and expression appeared to mature with age, peaking into early adulthood. Whilst GBP5 is a key gene involved in interferon signaling pathways in active TB, it is also upregulated in innate immune responses to a broad range of viral infections including influenza, and SARS-CoV-2.^30–33^ This may explain poor diagnostic discrimination. However, we did not observe a difference in GBP5 expression between non-TB meningoencephalitis and non-CNS infections. GBP5, therefore, may not be a good candidate gene for the diagnosis of TBM in children. The concept of the host immune response changing with age is consistent with faster progression of TB infection to disease or dissemination in young children <2 years of age.^34–36^

An exploratory analysis of the whole blood RNA sequence identified differentially expressed genes between children with TBM and other infections. The most frequently LASSO selected-genes have functions in epithelial integrity (TGM1),^37^ cellular metabolism within the electron transport chain (NDOR1)^38,39^ and protein trafficking/regulation (TXLNB).^40,41^ These genes have not been previously linked to TB pathogenesis and their biological relevance is uncertain. AUCs for these individual genes exceeded those within the original 3-gene host response signature, though are optimistic as analyses were limited to definite TBM without external validation. These preliminary signals should inform further analysis, rather than serve as diagnostic candidates. We plan to apply a prespecified machine-learning pipeline for robust gene selection, and validate a final signature in independent cohorts, before inferring diagnostic potential.

Study limitations include a single geographical setting, retrospective testing of archived samples, small cohort and possible case-control misclassification. The clinical reference standard comprised 55% unconfirmed (probable and possible) TBM cases, reflecting the lack of a perfect comparator, but is consistent with other studies.^42,43^ Misclassification of controls was mitigated by “recovery without ATT” criteria in children with presumptive meningoencephalitis. Clinical improvement without ATT excludes TBM, due to universal fatality without ATT, however, hospital stay may have been too short to be certain. One third of children with TBM received corticosteroids before enrollment, potentially affecting GBP5 expression. Diagnostic cutoffs were not explored as the 3-gene host response signature showed insufficient performance for clinical utility; such analysis is required in later biomarker development.

Study strengths include our well-characterized samples and the parallel evaluation of 3-gene host response signature in adults and children, which provided insights beyond diagnostic performance alone and reinforced the concept that host immune biomarkers used to diagnose TBM in children should not be extrapolated from adults. Stored whole blood in our study allows for high RNA yield, robust and reproducible expression data, such that exploration of novel gene sets to create pediatric-specific diagnostic signatures is possible.

## CONCLUSION

The 3-gene host response signature measured by RNA sequencing has poor ability to discriminate TBM from non-TBM in children. A pediatric-specific gene host signature for severe childhood TB with TBM may exist, but further development and validation are required.

## ACKNOWLEDGMENTS

*We thank parents/carers of children and adolescents who participated in this study and all the staff at participating sites. We also thank the independent members of the Trial Steering Committee: Victor Musiime, Janet Darbyshire, Peter Donald, Vidya Mave and Sabine Verkuijl for their review and approval of the manuscript.*

## Supplementary Material


