# Supplementary material for: The Utility of a Three-gene Host Response to Discriminate Tuberculous Meningitis From Other Infections in Children
Source: Pediatr Infect Dis J. 2025 Dec 23;45(5):466–74. doi: 10.1097/INF.0000000000005105 (PMC13064836; doi:10.1097/INF.0000000000005105)
Supplement: Supplementary file 1 [file inf-45-0466-s001.pdf]

SUPPLEMENTARY DIGITAL CONTENT 1. Uniform case definition for research - Definition of definite, probable, possible tuberculous meningitis

**Clinical entry criteria** – symptoms and signs of meningitis including one or more of headache, irritability, vomiting, fever, neck stiffness, convulsions, focal neurological deficits, altered consciousness or lethargy
**Tuberculous meningitis classification**<br>***Definite***<br>Patients fulfil criterion A or B<br>A) Clinical entry criteria plus one or more of the following: acid-fast bacilli seen on CSF; Mycobacterium tuberculosis cultures from CSF; or a CSF positive commercial nucleic acid amplification test<br>B) Acid-fast bacilli seen in the context of histological changes consistent with tuberculosis in the brain or spinal cord with suggestive symptoms or signs and CSF changes, or visible meningitis (on autopsy)<br>***Probable***<br>Clinical entry criteria plus a total diagnostic score of 10 or more points (when cerebral imaging is not available) or 12 or more points (when cerebral imaging is available) plus exclusion of alternative diagnoses. At least 2 points should come from CSF or cerebral imaging criteria.<br><br>***Possible***<br>Clinical entry criteria plus a total diagnostic score of 6-9 points (when cerebral imaging is not available) or 6-11 points (when cerebral imaging is available) plus exclusion of alternative diagnoses. Possible tuberculosis cannot be diagnosed or excluded without doing a lumbar puncture or cerebral imaging<br><br>***Not tuberculous meningitis***<br>Alternative diagnosis established, without a definitive diagnosis of tuberculous meningitis or other convincing signs of dual disease

Marais et al. Tuberculous meningitis: uniform case definition for use in clinical research Lancet Infect Dis 2010: 10: 803-12

SUPPLEMENTARY DIGITAL CONTENT 2. TBM severity grading as per British medical research council grade

**Grade**	**Definition**
Grade 1	GCS 15 and no focal neurology
Grade 2	GCS 11-14 or GCS 15 with focal neurology
Grade 3	GCS < 11 with or without focal neurology

SUPPLEMENTARY DIGITAL CONTENT 3. Diagnoses for non-CNS infections in the control group (n=13)

**Diagnosis**
Febrile seizure
Headache of unknown cause
Sepsis
Macrophage activation syndrome
Intracranial haemorrhage
Inborn error of metabolism
Cerebral palsy and bacteraemia
HIV
Congenital hydrocephalus with intercurrent illness
Lumbar spinal abscess
Viral illness unknown aetiology
Urinary tract infection
Autoimmune encephalitis

SUPPLEMENTARY DIGITAL CONTENT 4. Three gene expression (incorporating TBP instead of KLF2) and TB score in TBM compared to controls

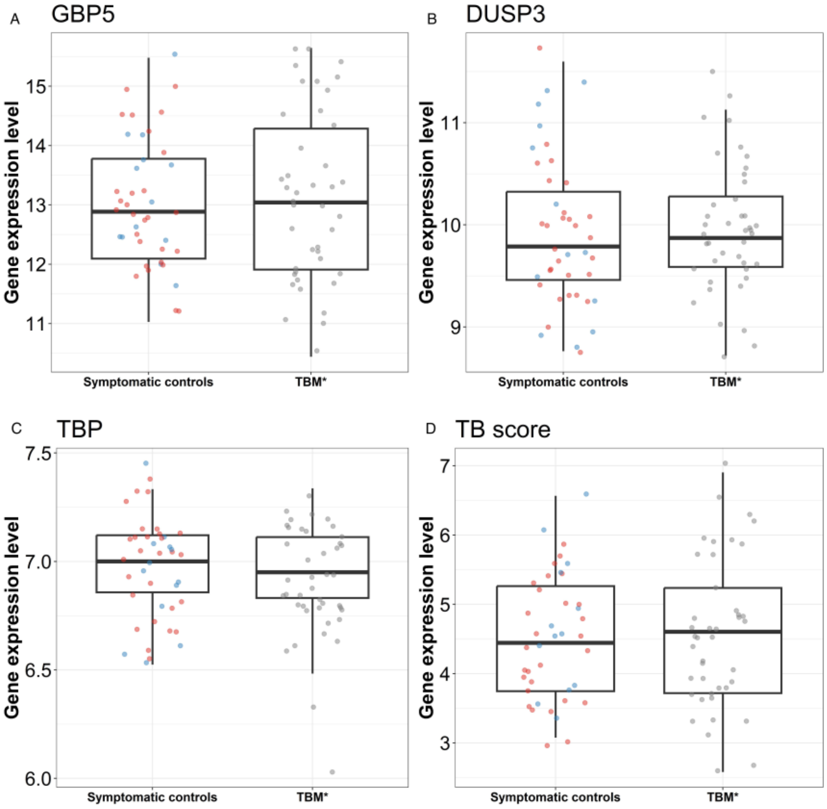

(A) GBP5 (B) DUSP3 (C) TBP (D) TB score. Comparisons of gene expression in TBM (n=42) and symptomatic controls (n=41). TBM* = all TBM. Controls were classified into other meningitis/encephalitis (n=28, red dots) or non-infectious (n=13, blue dots). Boxes indicate inter-quantile range, the horizontal line indicates the median, and dots indicate individual participant data. Comparisons were performed using Mann-Whitney U-test.

SUPPLEMENTARY DIGITAL CONTENT 5. Three gene expression and TB score in controls with meningoencephalitis compared to those without a CNS infection

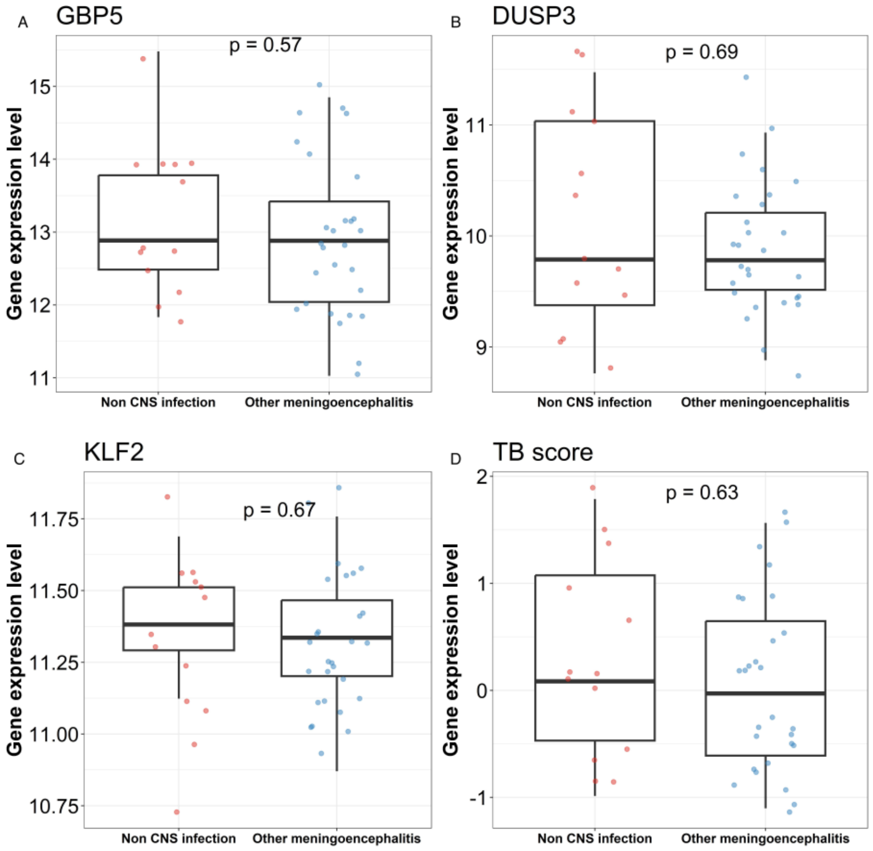

(A) GBP5 (B) DUSP3 (C) KLF2 (D) TB score. Comparisons of gene expression in meningoencephalitis caused by pathogens other than Mycobacterium tuberculosis (blue dots, n=28) and children with non CNS infections (red dots, n=13). Boxes indicate inter-quantile range, the horizontal line indicates the median, and dots indicate individual participant data. Comparisons were performed using Mann-Whitney U-test.

SUPPLEMENTARY DIGITAL CONTENT 6. Influence of corticosteroid therapy on 3-gene expression and TB score in children with TBM compared to controls

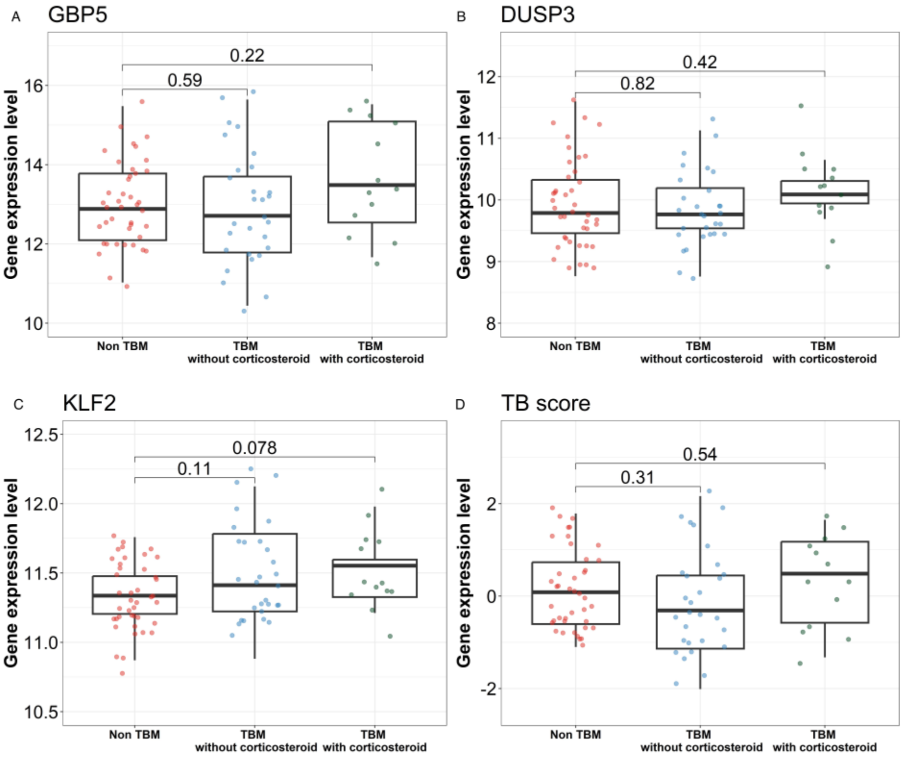

(A) GBP5 (B) DUSP3 (C) KLF2 (D) TB score. Comparisons of gene expression between children who received corticosteroid therapy prior to enrolment (green dots, n=13) and children who didn't receive corticosteroids therapy (blue dots, n=29) against controls (red dots, n=41). Boxes indicate inter-quantile range, the horizontal line indicates the median, and dots indicate individual participant data. Comparisons were performed using Mann-Whitney U-test.

SUPPLEMENTARY DIGITAL CONTENT 7. Baseline characteristics of adult 3 gene host signature study

	**TBM**<br>N = 281	**Other brain infections**<br>N = 50
**Age** (median, IQR)	41 (31, 54)	42 (30, 60)
**Male** (n, %)	183 (65%)	33 (66%)
**HIV**		
Negative (n, %)	207 (74%)	37 (100%)
Positive (n, %)	74 (26%)	0 (0%)
Unknown (n, %)	0	13
**Glasgow Coma Score** (median, IQR)	14 (12, 15)	11 (9, 13)
**TBM diagnostic category**		
Definite TBM (n, %)	134 (48%)	-
Probable TBM (n, %)	98 (35%)	-
Possible TBM (n, %)	49 (17%)	-
**Microbiological tests**		
MGIT culture positive (n,%)	91 (32%)	-
Xpert/Ultra positive (n,%)	81 (28.8 %)	-
Microscopy positive (n,%)	83 (30%)	-
**CSF parameters**		
leukocyte count (x 10^3 cells/ml)	139 (17, 350)	466 (114, 2,956)
lymphocytes (%)	89 (70, 100)	49 (10, 86)
neutrophils (%)	0 (0, 23)	50 (14, 90)
protein (g/L)	1.65 (0.97, 2.41)	1.19 (0.67, 3.91)
glucose (mmol/L)	2.57 (1.70, 3.50)	2.96 (0.74, 4.17)

SUPPLEMENTARY DIGITAL CONTENT 8. Diagnostic performance of TB score using TBP as a substitute for KLF-2 in TBM

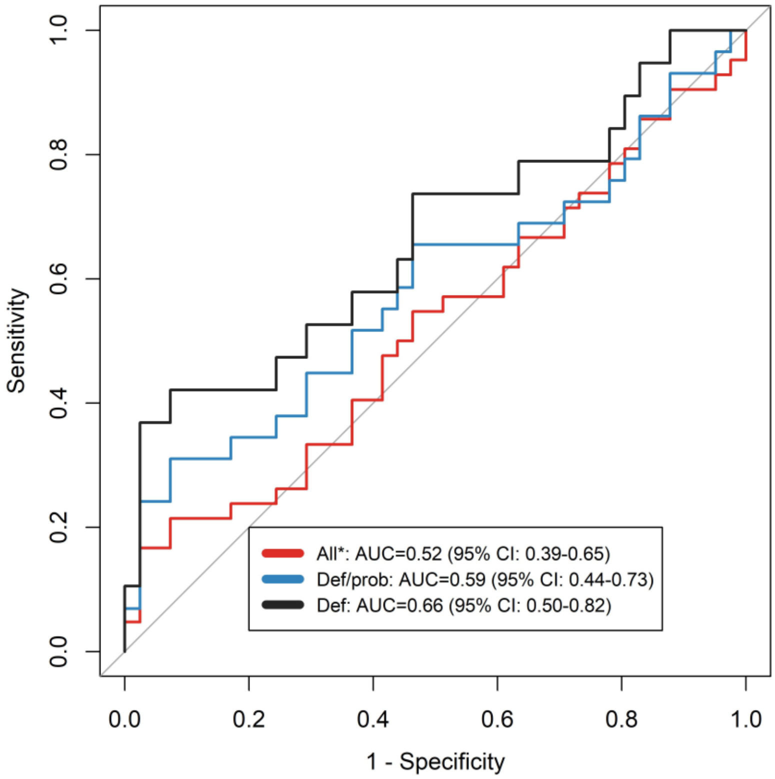

Receiver operating characteristics (ROC) curves for distinguishing all TBM (n=42), definite + probable TBM (n=29) and definite TBM (n=19) against symptomatic controls (n=41) using GBP5 + DUSP3/2- TBP to calculate TB score.

SUPPLEMENTARY DIGITAL CONTENT 9. The influence of TB lung on 3-gene expression and TB score

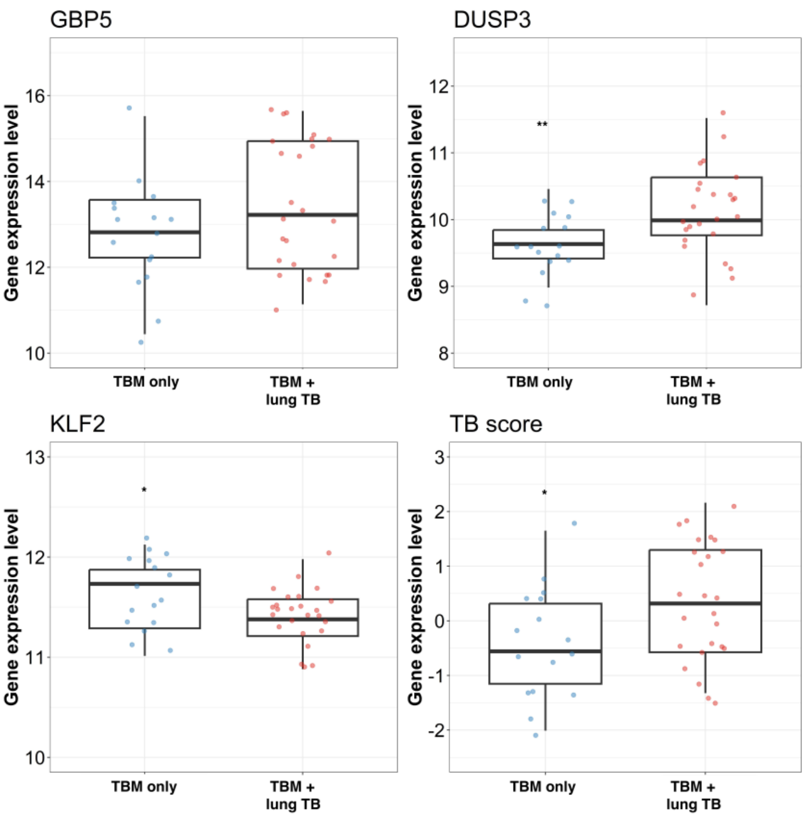

Each dot represents individual participant data. Boxes represent inter-quartile range and the horizontal line represents the median. (A) GBP5 (B) DUSP3 (C) KLF2 (D) TB score. TB in lung is defined by a composite clinical reference standard of microbiologically confirmed and presumptive pulmonary TB. TBM only in blue dots (n=16) and with TB in lung in red dots (n=25). TBM = definite/probable/possible tuberculous meningitis. Comparisons were performed using Mann-Whitney U-test. *P<0.05, **P<0.01

SUPPLEMENTARY DIGITAL CONTENT 10. Top three differentially expressed (DE) genes between children with definite TBM and other infections

A

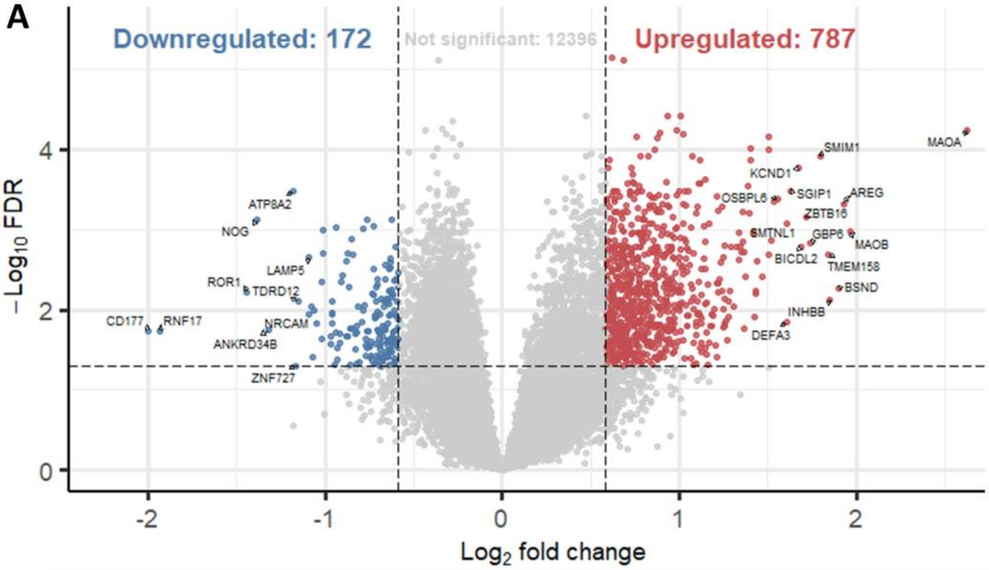

B

Gene	LASSO frequency	Regulation	LFC	FDR
TGM1	4579	↑	0.83	5.07×10^{-4}
TXLNB	4282	↑	0.72	3.29×10^{-4}
NDOR1	3680	↑	0.68	7.76×10^{-6}

(A) Volcano plot of differential gene expression. Each point represents a gene, plotted by $\log_2$ fold change (LFC) and –log10 FDR (false discovery rate) by Benjamini–Hochberg correction. DE genes were defined by thresholds of LFC > 0.585 and adjusted p < 0.05. In total, 959 DE genes were identified: 787 upregulated (red) and 172 downregulated (blue).
(B) The top three DE genes selected by a LASSO regression model across 10,000 bootstrap iterations, shown with their frequency of selection, regulation direction (up or down), log2 fold change, and FDR.

SUPPLEMENTARY DIGITAL CONTENT 11. The top 20 most frequently selected genes from a LASSO model across 10,000 bootstrapped iterations. Genes are shown in order of selection

frequency, together with log(2) fold change (LFC), false discovery rate (FDR), direction of regulation, and area under the receiver operating characteristic (ROC) curve (AUC) with corresponding 95% confidence intervals (CIs).

* AUC with 95% confidence intervals was calculated using DeLong's method
† ACO12651.1 corresponds to a reference genomic sequence for the human calpain-3 gene (CAPN3)

	Gene	Selection frequency	LFC	Adjusted FDR	Regulation direction	AUC*	95% CIs*
1	TGM1	4579	0.83	5.07x10(-4)	↑	0.814	0.694 - 0.934
2	TXLNB	4282	0.72	3.29x10(-4)	↑	0.780	0.635 - 0.926
3	NDOR1	3680	0.68	7.76x10(-6)	↑	0.828	0.708 - 0.948
4	AANAT	3424	1.40	9.69x10(-5)	↑	0.902	0.825 - 0.980
5	C9orf43	3357	-0.72	3.08x10(-3)	↓	0.791	0.661 - 0.921
6	STMP1	3164	0.94	3.75x10(-5)	↑	0.873	0.786 - 0.960
7	SGIP1	3044	1.63	3.29x10(-4)	↑	0.829	0.714 - 0.945
8	MAMLD1	3031	1.12	2.65x10(-4)	↑	0.846	0.742 - 0.950
9	ZNF844	2532	0.63	1.63x10(-3)	↑	0.819	0.689 - 0.949
10	MBNL3	2448	1.12	1.38x10(-4)	↑	0.847	0.748 - 0.947
11	CACNA1F	2331	0.59	5.55x10(-3)	↑	0.793	0.679 - 0.908
12	PTGDS	2208	0.60	2.42x10(-2)	↑	0.716	0.572 - 0.861
13	AJUBA	2188	-0.90	3.66x10(-3)	↓	0.769	0.647 – 0.891
14	ZNF205	2122	0.81	3.03x10(-3)	↑	0.787	0.665 – 0.908
15	NEURL1	1979	-0.78	9.91x10(-4)	↓	0.807	0.682 - 0.933
16	AREG	1934	1.93	4.75x10(-4)	↑	0.721	0.568 – 0.875
17	NUMBL	1723	1.03	6.43x10(-5)	↑	0.845	0.740 - 0.949
18	CHKB	1701	0.68	7.25x10(-4)	↑	0.806	0.688 - 0.924
19	JAZF1	1541	0.76	7.00x10(-5)	↑	0.823	0.716 – 0.930
20	AC012651.1 (CAPN3) †	1523	0.64	4.81x10(-3)	↑	0.782	0.663 - 0.901
